# Three Pilot Randomized Controlled Trials Evaluating a Persuasive Health Communication Intervention for Adult Emergency Department Patients Declining HIV/HCV Testing

**DOI:** 10.29024/jsim.206

**Published:** 2024-10-07

**Authors:** ROLAND C. MERCHANT, MELISSA A. CLARK, MICHAEL P. CAREY, TAO LIU, GEORGE LOO, ETHAN A. COWAN

**Affiliations:** Department of Emergency Medicine, Icahn School of Medicine at Mount Sinai, US; Department of Health Services, Policy and Practice, School of Public Health, Brown University, US; Department of Behavioral and Social Sciences, School of Public Health, Brown University, US; Department of Biostatistics, School of Public Health, Brown University, US; Department of Emergency Medicine, Icahn School of Medicine at Mount Sinai, US; Department of Population Health Science and Policy, Icahn School of Medicine at Mount Sinai, US; Department of Emergency Medicine, Icahn School of Medicine at Mount Sinai, US

**Keywords:** HIV testing, hepatitis C, emergency service, hospital, persuasive communication

## Abstract

**Background:**

The lack of evidence-based interventions to overcome patient refusal limits the success of emergency department (ED)-based HIV and hepatitis C virus (HCV) testing for diagnostic purposes or screening. We created a persuasive health communication intervention (PHCI) designed to overcome ED patient reluctance to accept HIV/HCV testing. In three pilot randomized controlled trials (pRCTs), we evaluated the performance of the PHCI when delivered by video or in-person by an HIV/HCV counselor, and as compared to a control condition video.

**Methods:**

Adult ED patients who declined HIV/HCV screening were enrolled. Participants were randomly assigned (1:1 allocation) in each pRCT as follows: pRCT 1: PHCI video vs. control condition video; pRCT 2: PHCI delivered in-person by HIV/HCV counselor vs. control condition video; and pRCT 3: PHCI delivered in-person by HIV/HCV counselor vs. the PHCI video. The primary outcome for each pRCT was acceptance of HIV/HCV testing post-intervention.

**Results:**

Acceptance of HIV, HCV or both tests post-intervention was: pRCT 1: PHCI video (n = 27) vs. control condition video (n = 28), 29.6% vs. 10.7%; p = 0.08; pRCT 2: PHCI delivered in-person by HIV/HCV counselor (n = 30) vs. control condition video (n = 30), 10.0% vs. 26.7%; p = 0.09; and pRCT3: PHCI delivered in-person by HIV/HCV counselor (n = 29) vs. the PHCI video (n = 29), 48.3% vs. 34.5%; p = 0.29.

**Conclusions:**

The results from these pRCTs are encouraging. ED patients who initially declined HIV/HCV testing can be persuaded instead to be screened for these infections. The PHCI, whether delivered in-person by an HIV/HCV counselor or video, is a promising intervention to encourage screening for these infections.

## INTRODUCTION

A continued problem limiting the success of emergency department (ED)-based HIV and hepatitis C virus (HCV) testing is testing refusal [1–5]. Patients refuse HIV/HCV testing whether it is for diagnostic purposes or for screening, and whether or not an opt-in, opt-out, or other approach is employed. Previous research has reported that adult ED patients at risk for, or later diagnosed with, HIV or HCV had initially declined ED-based testing [6]. We have not identified any existing evidence-based interventions to persuade adult ED patients who decline HIV/HCV testing to agree to be tested. ED clinicians lack published guidance on effective approaches to overcome patient reluctance to be tested. As a result, HIV/HCV diagnoses go unrecognized, leading to continued spread of these infections, and potentially higher morbidity and mortality among those diagnosed later in the course of their infection.

With the assistance of 44 stakeholders (ED medical staff, HIV/HCV counselors, and English- or Spanish-speaking ED patients), we created a persuasive health communication intervention (PHCI) designed to overcome ED patient reluctance to accept HIV/HCV testing [7]. We planned to evaluate its efficacy in convincing ED patients who initially declined HIV/HCV testing to agree to be tested for these infections. We were also interested in assessing if the PHCI was equally or more efficacious when delivered in-person by an HIV/HCV test counselor, as compared to video-based delivery. The rationale for videos as comparators was due to their advantages over in-person delivery of an intervention, including providing content in a uniform manner, enabling presentation of the intervention at any time and to multiple patients in parallel, and potentially reducing burdens on ED staff time. In addition, to test the value of the PHCI itself, we wanted to know if the PHCI, either delivered in-person or by video, was more efficacious than a control condition video.

We prepared two videos for this investigation. The first video was a time-matched, three-minute control condition video that contained content solely from United States (US) Centers for Disease Control and Prevention (CDC) testing brochures. The CDC control condition video was designed as an information-only, passive “standard of care” mode of providing CDC-recommended information to people being tested for HIV/HCV. The second video depicted two actors using the PHCI (PHCI video). One actor portrayed a physician delivering the PHCI, and the other actor portrayed an ED patient who had initially declined HIV/HCV testing. By the end of the PHCI video, the patient agrees to be tested for HIV/HCV. This PHCI video permitted us to assess whether the PHCI was more efficacious when delivered in person or by video. It also enabled a test of whether receiving an intervention directly or watching it being delivered to someone else resulted in greater acceptance of HIV/HCV testing.

We evaluated the three interventions (PHCI in-person by HIV/HCV counselor, PHCI video, or CDC control condition video) in three separate, pilot randomized, controlled trials (RCTs) among adult ED patients who declined simulated opt-out HIV/HCV screening. In the first pRCT, we compared the PHCI video to the CDC control condition video. In the second pRCT, we compared the PHCI when delivered in-person by an HIV/HCV counselor to the CDC control condition video. In the third pRCT, we compared the PHCI when delivered in-person by an HIV/HCV counselor to the PHCI video.

The primary purpose of this investigation was to estimate effect sizes resulting from comparing the three interventions (PHCI in-person by HIV/HCV counselor, PHCI video, or CDC control condition video) by their efficacy of convincing ED patients to be tested for HIV/HCV. We planned for the intervention effect sizes to inform the study design and sample size for a subsequent larger RCT evaluating the PHCI. The primary outcome measured was post-intervention acceptance of testing for HIV, HCV or both infections. In addition, we explored if the reasons participants cited for declining HIV/HCV testing differed by the type of intervention they received (PHCI in-person by HIV/HCV counselor, PHCI video, or CDC control condition video). If reasons for declining differed by intervention, this information could assist in subsequent revisions of an intervention. We also explored if acceptance or decline of HIV/HCV testing was related to participant-reported HIV/ HCV risk factors, which would suggest participants might be basing their decision to be tested on their self-perception of likelihood of having these infections. To obtain information on the need to revise the interventions, we examined how participants perceived the interventions they received were convincing, motivating and respectful of decision-making.

## METHODS

### STUDY DESIGN AND SETTING

This investigation consisted of three pRCTs among adult patients at two different EDs. pRCTs 1 and 2 were performed at an urban tertiary care Level 1 trauma center, academic “safety net” hospital [8] with over 110,000 annual adult patient visits. HIV/HCV testing is performed at the clinician’s discretion in this hospital’s ED. pRCT 3 was performed at an urban academic “safety net” hospital [8] with over 75,000 annual adult patient visits. The ED at this hospital has an ongoing, non-targeted, universal HIV/HCV screening program. Each hospital’s institutional review board approved the study.

We originally planned for 100 participants, 50 participants/study arm for each pRCT (n = 300 across all three pRCTs). Due to the COVID-19 pandemic, we had to reduce the anticipated sample size to a maximum of 60 participants, 30 participants/study arm for each pRCT (n = 180 across all three pRCTs).

### PHCI CONTENT

Development of the PHCI has been described previously [7]. In brief, the study authors prepared an initial PHCI script using six successive components (information, education, gain, loss, common concerns, and call to action). The PHCI was crafted to be very brief (2–3 minutes in length); could be delivered orally in person in a clinical setting with minimal training; presents unambiguous, clear and concise arguments to convince an ED patient who initially declined HIV/HCV testing instead to decide to be tested for these infections; and is respectful of patient autonomy and decision-making. PHCI content was informed by key constructs from health behavior change and message design theories (Health Belief Model, Extended Parallel Process Model, and Prospect Theory) [9–13]. The PHCI purposely did not engage patients in assessments about their risk for having or acquiring HIV/HCV. This purposeful omission was based on our and other research that ED patients often misattribute or discount their HIV/HCV risk [14, 15] and frequently decline screening out of a likely incorrect belief that they are not at risk [1, 16], and also our research that HIV/HCV risk assessment and feedback behavioral interventions did not increase HIV/HCV screening uptake [2–4].

**Figure 1:**
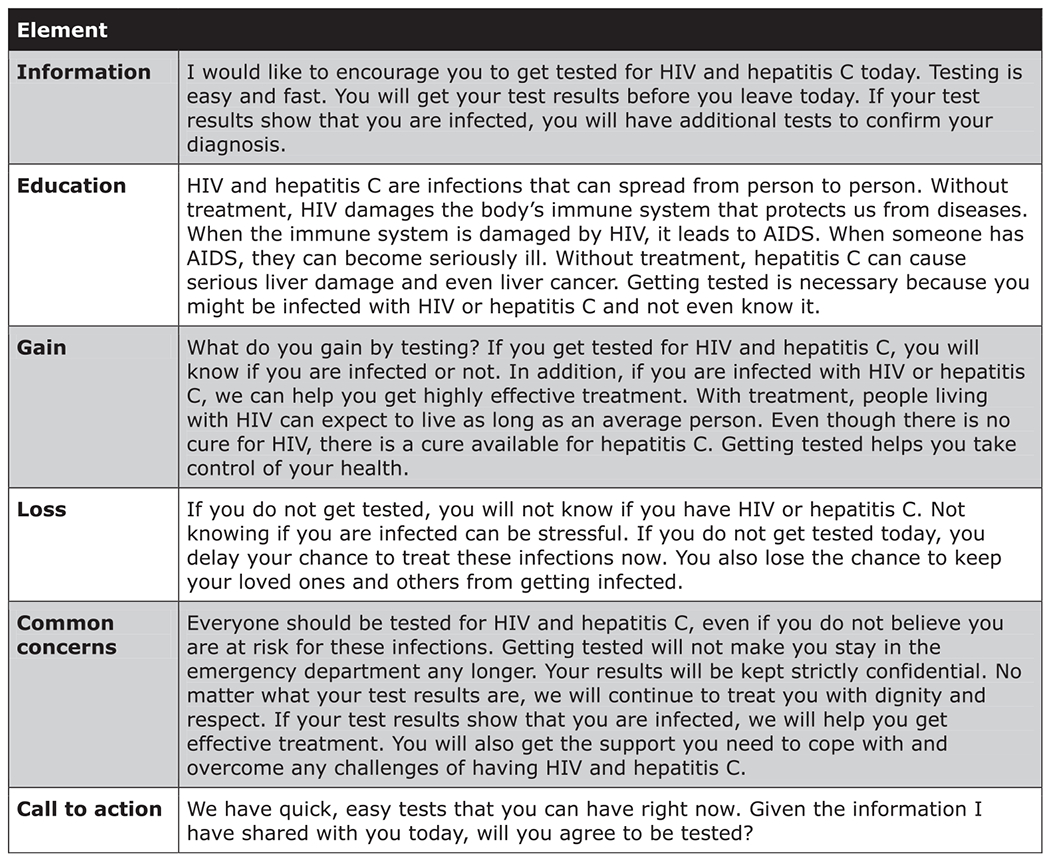
Persuasive Health Communication Intervention content.

The PHCI was presented to 44 stakeholders (12 English- and 12 Spanish-speaking adult ED patients who had declined HIV/HCV screening; 15 ED medical staff (attending and resident physicians, nurse practitioners or physician assistants), and five HIV/HCV counselors. Each stakeholder was interviewed using a semi-structured interview script to solicit their perspectives on how convincing, clear, complete and respectful the PCHI components were, and how the PHCI could be improved. The PHCI content was revised in an iterative manner based on the interview results. The final PHCI is shown in Figure 1.

### PHCI VIDEO

The PHCI video depicts an actor portraying a female physician delivering the PHCI in person to a male actor portraying ED patient who had declined HIV/HCV screening. By the end of the PHCI video, the patient agrees to be tested for HIV/HCV. The behavioral theories that help explain the PHCI video’s efficacy in persuading ED patients to be tested for HIV/HCV are as follows. Exemplification theory [17] argues that viewing the experience of the patient depicted in the video is likely to be effective because of the vivid and impactful nature of the personal interaction. Social cognitive theory [18] holds that persons are able to model behavior through observational learning; having the actor in the PHCI video agree to be tested for HIV/HCV demonstrates the behavior we wish patients to emulate.

### CDC CONTROL CONDITION VIDEO

**Figure 2:**
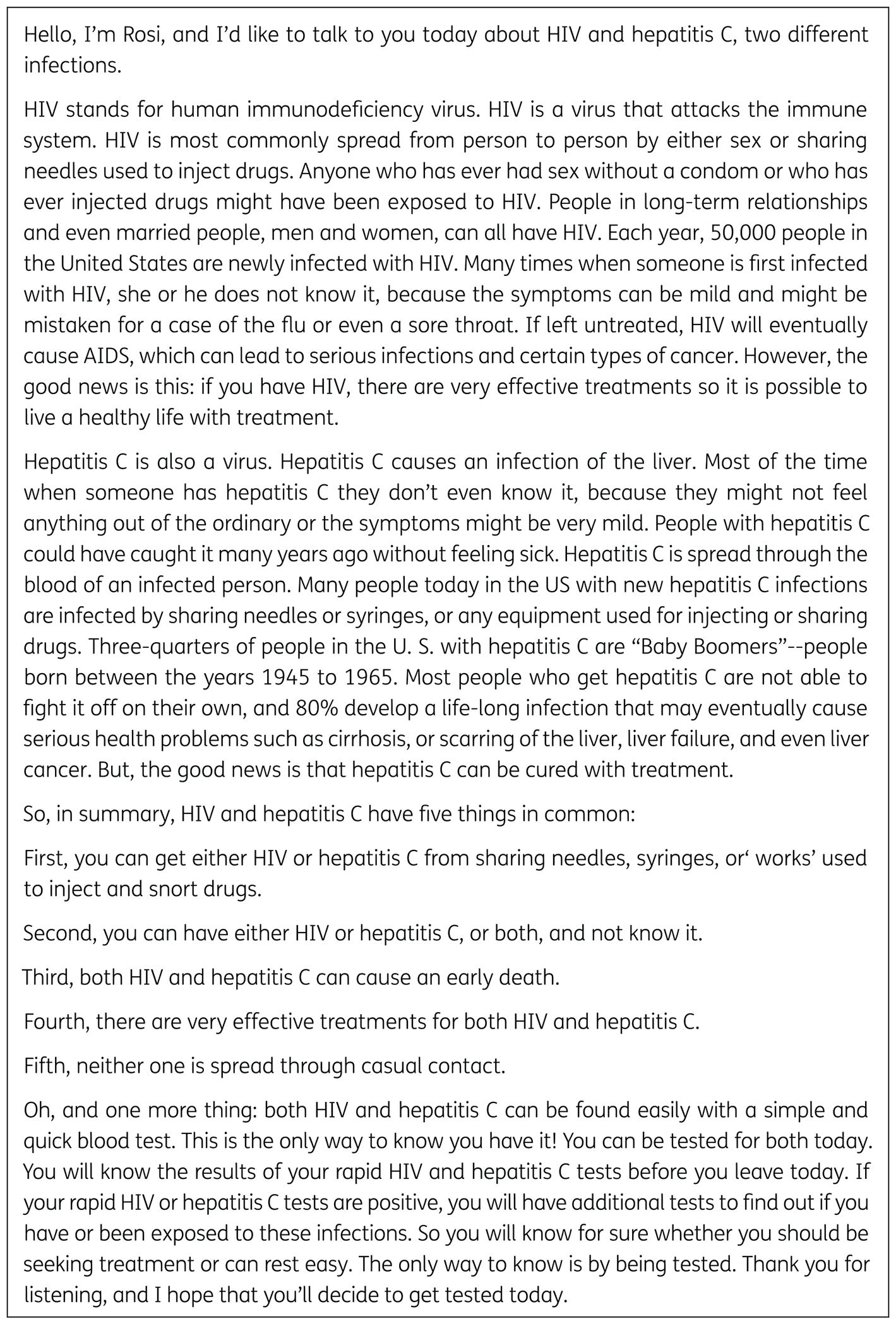
Centers for Disease Control and Prevention (CDC) Control Condition Video Script.

We derived the three-minute CDC control condition video script from CDC informational brochures about HIV and HCV testing (Figure 2) [19, 20]. We made minor edits to the brochures so they could be combined into a single video script. The video briefly describes HIV and HCV, their transmission modes, and reasons for testing. The video (English and Spanish language versions) featured the same performer talking directly to viewers using the script via a teleprompter. Basic, non-intrusive graphics emphasizing the main points from the script were displayed simultaneously with the video message. Of note, the performer in the CDC control condition video was the same actor who portrayed the female physician in the PHCI video.

### STUDY SAMPLE

ED patients were study eligible if 18–64-years-old; English- or Spanish-speaking; not critically ill or injured; not prison inmates, under arrest, nor undergoing home confinement; not presenting for an acute psychiatric illness; not intoxicated; and did not have a physical or cognitive impairment that prevented them from providing consent or participating in the study. Excluded also were patients who were already known to be HIV or HCV infected, taking HIV pre-exposure prophylaxis (PrEP), participating in HIV vaccine or other HIV/HCV research studies, or had been tested for HIV or HCV within the prior year.

### PARTICIPANT RECRUITMENT AND SIMULATED OPT-OUT HIV/HCV SCREENING

HIV/HCV counselors serving as research assistants reviewed the electronic health records (EHRs) of patients present in the ED during data collection periods to assess which patients were potentially study eligible. The HIV/HCV counselors recorded the demographic characteristics of all ED patients whose EHRs they reviewed for study eligibility. HIV/HCV counselors approached those who appeared to meet study eligibility criteria by the EHR review. The HIV/HCV counselors initiated simulated opt-out rapid HIV/HCV screening, using the following script: “Please tell me what you would say or do if I said the following: ‘As part of a study, we are offering a random sample of patients free rapid testing for HIV and hepatitis C. HIV and hepatitis C testing is recommended by medical professionals. I would like to test you for these two infections, unless you tell me that you do not want to be tested. The test is free and you will get the test results while you are still here in the emergency department. If the test shows that you could be infected, I will help you get the care you need.’ If I said that to you, would you get tested or would you instead say that you did not want to be tested?”

For patients who indicated that they would be tested, the HIV/HCV counselors explained that the intention of the study was to find people who declined to be tested. These patients were thanked for their time, provided a brochure of local resources about testing options, and informed that they may ask their ED clinician about testing. For patients who indicated that they would not be tested, the HIV/HCV counselors would confirm study eligibility by asking about their HIV/HCV testing history. Afterwards, the HIV/HCV counselors invited eligible individuals to participate in the study, explained the purpose and steps involved in the study, and asked them to consent to participate.

### STUDY PROCEDURES

In pRCT 1, participants were randomly assigned (1:1 allocation) to the PHCI video or the CDC control condition video. In pRCT 2, random assignment (1:1 allocation) was to HIV/HCV counselor in-person delivery of the PHCI or to the CDC control condition video. In pRCT 3, random assignment (1:1 allocation) was to HIV/HCV counselor in-person delivery of the PHCI or to the PHCI video.

Post-intervention--after completion of the video or the terminal part of the PHCI--the HIV/HCV counselor asked participants to be tested for HIV and HCV: “We have quick, easy tests for HIV and hepatitis C that you can have right now. Given the information I have shared with you today, will you agree to be tested?” Participants could respond that they would be tested for both HIV and HCV, either test, or neither test. Those who did not agree to testing for either HIV, HCV or both HIV and HCV were encouraged to be tested in the future and provided with resources for local HIV/HCV testing, and informed that they later could ask the ED medical staff to test them as part of their clinical care. Finally, HIV/HCV counselors asked them to indicate their main reason for declining HIV/HCV testing.

Among those who agreed to HIV/HCV testing for pRCT 1 and 2, the HIV/HCV counselors performed fingerstick rapid HIV/HCV testing. For pRCT 3, samples for HIV/HCV testing were obtained by ED nursing staff by phlebotomy, and conventional testing was performed by the hospital laboratory. All participants, whether tested or not, were asked to complete an audio computer-assisted self-interviewer (ACASI) questionnaire about their risk factors for HIV/HCV infections. They also were asked to rate how convincing, motivating and respectful of decision-making they perceived the intervention type they received (PHCI in-person by HIV/HCV counselor, PHCI video, or CDC control condition video) were on a five-point scale (e.g., “not at all” to “very” convincing). Participants who completed the questionnaire were provided with a gift card.

### ANALYSES

Participants in each study arm were compared according to their demographic characteristics. HIV/HCV test acceptance post-intervention was compared by study arm within each pRCT. Fisher’s exact testing was performed for these comparisons because of the small sample sizes. Reasons for declining HIV/HCV testing after the intervention also were compiled by study arm within each pRCT. Reported HIV/HCV risk factors and participant perceptions of the intervention they received (convincing, motivating, respectful of decision-making) were stratified by acceptance or decline of HIV/HCV testing post-intervention. Reasons for declining HIV/HCV testing, reported HIV/HCV risk factors, and participant perceptions were not compared using statistical testing due to small numbers of participants in each category.

## RESULTS

### STUDY POPULATION

The number of participants completing each pRCT were as follows: pRCT 1 (CDC control condition video vs. PHCI video) n = 55, pRCT 2 (CDC control condition video vs. counselor-delivered PHCI) n = 60, and pRCT 3 (PHCI video vs. counselor-delivered PHCI) n = 58. Comparison of demographic characteristics of participants for each pRCT by study arm are provided in Table 1. Demographic characteristics were similar except for slightly more people who were single or married/coupled in the PHCI video arm and more who were divorced in the CDC control condition video arm in pRCT 1.

### POST-INTERVENTION HIV/HCV TESTING ACCEPTANCE, REASONS FOR DECLINING HIV/HCV TESTING AND SELF-REPORTED HIV/HCV RISK

Table 2 shows the results for each pRCT from comparing the proportions of participants accepting HIV/HCV testing post-intervention. Although participants were offered both HIV and HCV testing, a minority in each pRCT accepted only HCV testing and thus declined HIV testing. None wanted only to be tested for HIV, and not HCV. Although not reaching statistical significance at the α = 0.05 level, acceptance of HIV, HCV or both testing was more frequent for the PHCI video than the CDC control condition video arm (24.8% vs. 12.5%) (pRCT 1); the CDC control condition video than the HIV/HCV counselor-delivered PHCI arm (26.7% vs. 10.0%) (pRCT 2); and the HIV/HCV counselor-delivered PHCI than the PHCI video a (48.3% vs. 34.5%) (pRCT 3).

Acceptance of testing for HIV, HCV or both by pRCT, regardless of intervention received, was: pRCT 1 (n = 55) 20.0%; pRCT 2 (n = 60) 18.3%; and pRCT 3 (n = 58) 41.4%. Acceptance of testing for HIV, HCV or both by intervention received was: CDC control condition video (n = 58) 19.0%; PHCI video (n = 56) 32.1%; and PHCI delivered by an HIV/HCV counselor (n = 59) 28.8%.

Consistent with prior research on ED-based HIV/HCV testing, the predominant reason for declining HIV or HCV testing in all three pRCTs and study arms was due to self-perceived lack of risk for having these infections (Table 3). Perception of lack of risk was cited by participants in the CDC control video arm than the HIV/HCV counselor-delivered PHCI arm in pRCT 2 as the reason for declining testing. Regarding self-reported HIV/HCV risk, condomless sex was reported frequently among participants in all pRCTs, particularly among those who declined HIV testing (Table 4). Although few participants across all pRCTs reported any HCV risk factors, the most frequently reported were a history of incarceration or inhalation drug use.

### PARTICIPANT ASSESSMENT OF INTERVENTION

Table 5 indicates how convincing, motivating or respectful participants rated the intervention they received (CDC control condition video, PHCI video, HIV/HCV counselor-delivered PHCI) in each pRCT, as stratified by acceptance of HIV/HCV testing. There were no clear patterns of differences for these ratings by testing acceptance.

The majority of participants in each pRCT indicated that they found the intervention they received was convincing (pRCT 1 (n = 55): 60.0%; pRCT 2 (n = 60): 71.7%; and pRCT 3 (n = 58): 69.0%); motivating (pRCT 1 (n = 55): 60.0%; pRCT 2 (n = 60): 70.0%; and pRCT 3 (n = 58): 58.6%); and respectful of patient decision-making autonomy (pRCT 1 (n = 55): 78.2%; pRCT 2 (n = 60): 86.7%; and pRCT 3 (n = 58): 87.9%). Participant assessments for each intervention across all pRCTs also were favorable: CDC control condition video (n = 58; convincing 64.0%; motivating 58.6%; and respectful 77.6%); PHCI video (n = 56; convincing 62.5%; motivating 55.4%; and respectful 83.9%); and HIV/HCV counselor-delivered PHCI (n = 59; convincing 74.6%; motivating 74.6%, and respectful 91.5%).

## DISCUSSION

Interventions to persuade ED patients who decline HIV/HCV testing should be: (1) brief and easy to administer and remember (essential for busy ED settings and competing demands faced by ED staff); (2) similar to routine clinical practice (permitting ED clinicians to adopt it easily and mimic their common practice of persuading reluctant patients to be tested, undergo procedures, and receive treatments); (3) theoretically-grounded (targeting the determinants of patient decision-making); and (4) supported by research (to maximize the intervention’s likely feasibility, acceptability, and efficacy). Believing that our PHCI has these attributes, we sought to estimate its efficacy through these three pRCTs. We anticipated that the PHCI’s six successive components (information, education, gain, loss, common concerns, and call to action) would better convince and motivate ED patients to agree to be tested for HIV/HCV, as compared to the passive informational CDC control condition video. We were unsure which method of delivery of the PHCI (counselor, in-person vs. video) would confer greater acceptance of HIV/HCV testing.

We found that after any of the three interventions (CDC control condition video, PHCI in-person or video) were delivered, participants agreed to be tested for HIV, HCV or both. This finding suggests that all interventions were efficacious in persuading these ED patients who initially declined to instead be tested for these infections. However, there were variations in testing acceptance for the same intervention across the pilot studies. The CDC control condition performed less well against the PHCI video, but better than the PHCI delivered by the HIV/HCV counselor. Yet, the PHCI video performed less well against the PHCI delivered by the HIV/HCV counselor. The small sample sizes preclude concluding which intervention performs best, or could be used as part of HIV/HCV screening or testing programs. However, we obtained data that can inform the plans for a subsequent larger, fully-powered RCT that can further examine which intervention might work best to convince ED patients to be tested for HIV/HCV.

There is scant research examining behavioral interventions to encourage ED patients who had declined HIV and/or HCV testing to be tested for these infections. Ubhayakar, et al. assessed a non-randomly assigned, single-arm intervention designed to increase testing uptake as part of a targeted ED HIV testing program [21]. Of 199 approached for HIV testing, 47% declined. Of these 94 decliners, 60 (64%) agreed to undergo a formal risk assessment with an HIV counselor involving a questionnaire-driven interview with a plan for risk reduction. After this intervention, 6.7% of the 60 decliners agreed to be tested. In contrast, our intervention, the PHCI we employed in this study, whether delivered by video or in-person by an HIV/HCV counselor, was not linked to reported HIV/HCV risk-taking behaviors. Although not directly comparable, post-intervention acceptance of HIV/HCV testing was higher for the interventions employed in this study across all pRCTs: CDC control condition video 19.0%; PHCI video 32.1%; and PHCI delivered by an HIV/HCV counselor 28.8%. These results are particularly encouraging for the PHCI as an intervention to increase ED patients’ acceptance of HIV/HCV screening.

There are several limitations to this investigation which was *a priori* a pilot study designed to obtain preliminary data on the efficacy of the interventions. The small sample size precluded robust conclusions in comparing interventions as well as statistical comparisons of the reasons for testing decline, HIV/HCV risk factors, and participant perceptions about the interventions they received. The first two pRCTs were conducted at a single institution while the third pRCT was performed at a different ED. Third, the COVID-19 pandemic not only limited the sample size available for this study, but might have influenced testing uptake in the ED, as well as who presented for care during this period. As such, generalization of findings to other EDs or time periods should be made cautiously.

## CONCLUSION

The PHCI, whether delivered in-person by an HIV/HCV counselor or video, persuaded ED patients to be tested for these infections. Although a larger RCT will assist in further evaluating the efficacy of the PHCI and how it can be delivered optimally, these results are encouraging and indicate that ED patients who initially declined HIV/HCV testing can be persuaded to be screened for these infections.

## Figures and Tables

**Table 1:** Demographic Characteristics of Participants in Three Pilot Randomized Controlled Trials.

	*pRCT 1 (n = 55)*			*pRCT 2 (n = 60)*			*pRCT 3 (n = 58)*		
	CDC CONTROL CONDITION VIDEO	PHCI VIDEO	*p-VALUE*	CDC CONTROL CONDITION VIDEO	HIV/HCV COUNSELOR-DELIVERED PHCI	*p-VALUE*	PHCI VIDEO	HIV/HCV COUNSELOR-DELIVERED PHCI	*p-VALUE*
	*n = 28*	*n = 27*	*p =*	*n = 30*	*n = 30*	*p =*	*n = 29*	*n = 29*	*p =*
***Demographic characteristics***									
**Median age in years (IQR)**	49 (38–57)	46 (34–52)	0.27	50 (39–56)	45 (34–53)	0.22	33 (26–45)	36 (28–56)	0.29
	# (%)	# (%)		# (%)	# (%)		# (%)	# (%)	
**Gender**									
Male	12 (42.9)	16 (59.3)	0.22	15 (50.0)	8 (26.7)	0.06	14 (48.3)	13 (44.8)	0.79
Female	16 (57.1)	11 (40.7)		14 (46.7)	22 (73.3)		15 (51.7)	16 (55.2)	
Transgender Female/Male-to-Female	0	0		0	0		0	0	
Transgender Male/Female-to-Male	0	0		1 (3.3)	0		0	0	
**Race/Hispanic ethnicity**									
White, non-Hispanic	17 (63.0)	18 (69.2)	0.79	23 (76.7)	20 (66.7)	0.39	10 (34.5)	14 (48.3)	0.28
Black/African-American, non-Hispanic	7 (26.0)	5 (19.2)		5 (16.7)	4 (13.3)		2 (6.9)	5 (17.2)	
Other, non-Hispanic	1 (3.7)	2 (7.7)		0	0		1 (3.5)	0	
Hispanic	2 (7.4)	1 (3.9)		2 (6.7)	5 (16.7)		14 (48.3)	7 (24.1)	
Asian	0	0		0	1 (3.3)		2 (6.9)	2 (6.9)	
American Indian or Alaskan Native	0	0		0	0		0	1 (3.5)	
**Language spoken**									
English	27 (100.0)	26 (100.0)	N/A	29 (96.7)	28 (93.3)	>0.99	29 (100)	29 (100)	N/A
Spanish	0	0		1 (3.3)	2 (6.7)		0	0	
**Partner status**									
Single	7 (25.9)	11 (42.3)	0.03	12 (40.0)	12 (40.0)	0.06	16 (55.2)	20 (69.0)	0.30
Married/Coupled	8 (29.6)	9 (34.6)		12 (40.0)	17 (56.7)		7 (24.1)	6 (20.7)	
Legally separated	1 (3.7)	1 (3.9)		1 (3.3)	0		1 (3.5)	1 (3.5)	
Divorced	11 (40.7)	2 (7.7)		5 (16.7)	0		0	1 (3.5)	
Widowed	0	2 (7.7)		0	0		1 (3.5)	1 (3.5)	
Not provided	0	1 (3.9)		0	1 (3.3)		4 (13.8)	0	
**Health care insurance**									
Private	11 (40.7)	14 (53.9)	0.79	13 (43.3)	16 (53.3)	0.69	14 (48.3)	12 (41.4)	0.28
Governmental	1 (3.7)	1 (3.9)		5 (16.7)	2 (6.7)		0	4 (13.8)	
Private and Governmental	11 (40.7)	7 (26.9)		8 (26.7)	9 (30.0)		10 (34.5)	10 (34.5)	
None	4 (14.8)	3 (11.5)		4 (13.3)	3 (10.0)		4 (13.8)	3 (10.3)	
Not provided	0	1 (3.9)		0	0		1 (3.5)	0	

Key: pRCT (pilot randomized controlled trial), CDC (Centers for Disease Control and Prevention, PHCI (persuasive health communication intervention), IQR (Interquartile Range).

**Table 2:** Post-Intervention Participant Acceptance of HIV or HCV Testing in each Pilot Randomized Controlled Trial.

	*pRCT 1 (n = 55)*			*pRCT 2 (n = 60)*			*pRCT 3 (n = 58)*		
	CDC CONTROL CONDITION VIDEO	PHCI VIDEO	*p-VALUE*	CDC CONTROL CONDITION VIDEO	HIV/HCV COUNSELOR-DELIVERED PHCI	*p-VALUE*	PHCI VIDEO	HIV/HCV COUNSELOR-DELIVERED PHCI	*p-VALUE*
	*n = 28*	*n = 27*		*n = 30*	*n = 30*		*n = 29*	*n = 29*	
	# (%)	# (%)	*p =*	# (%)	# (%)	*p =*	# (%)	# (%)	*p =*
**One or both tests acceptance**									
Accepted both HIV and HCV testing	3 (10.7)	6 (22.2)	0.15	5 (16.7)	3 (10.0)	0.15	6 (20.7)	10 (34.5)	0.53
Accepted only HIV testing	0	0		0	0		0	0	
Accepted only HCV testing	0	2 (7.4)		3 (10.0)	0		4 (13.8)	4 (13.8)	
Refused HIV and HCV testing	25 (89.3)	19 (70.4)		22 (73.3)	27 (90.0)		19 (65.5)	15 (51.7)	
**Any test acceptance**									
Accepted HIV, HCV or both testing	3 (10.7)	8 (29.6)	0.08	8 (26.7)	3 (10.0)	0.09	10 (34.5)	14 (48.3)	0.29
Refused HIV, HCV or both testing	25 (89.3)	19 (70.4)		22 (73.3)	27 (90.0)		19 (65.5)	15 (51.7)	

Key: pRCT (pilot randomized controlled trial), CDC (Centers for Disease Control and Prevention), HCV (hepatitis C virus), PHCI (persuasive health communication intervention).

**Table 3:** Reasons Participants Cited for Declining HIV or HCV Testing in each Pilot Randomized Controlled Trial.

	*pRCT 1 (n = 55)*				*pRCT 2 (n = 60)*				*pRCT 3 (n = 58)*			
	CDC CONTROL CONDITION VIDEO		PHCI VIDEO		CDC CONTROL CONDITION VIDEO		HIV/HCV COUNSELOR-DELIVERED PHCI		PHCI VIDEO		HIV/HCV COUNSELOR-DELIVERED PHCI	
REASONS FOR DECLINING HIV TESTING, # (%)	ACCEPTED HIV AND/OR HCV TESTING	DECLINED HIV AND/OR HCV TESTING	ACCEPTED HIV AND/OR HCV TESTING	DECLINED HIV AND/OR HCV TESTING	ACCEPTED HIV AND/OR HCV TESTING	DECLINED HIV AND/OR HCV TESTING	ACCEPTED HIV AND/OR HCV TESTING	DECLINED HIV AND/OR HCV TESTING	ACCEPTED HIV AND/OR HCV TESTING	DECLINED HIV AND/OR HCV TESTING	ACCEPTED HIV AND/OR HCV TESTING	DECLINED HIV AND/OR HCV TESTING
	*n = 3*	*n = 25*	*n = 8*	*n = 19*	*n = 8*	*n = 22*	*n = 3*	*n = 27*	*n = 4*	*n = 19*	*n = 4*	*n = 15*
I do not believe that I am at risk for HIV	0	15 (60.0)	2 (25.0)	12 (63.2)	2 (25.0)	17 (77.3)	0	11 (40.7)	4 (100.0)	17 (89.5)	4 (100.0)	11 (73.3)
I do not feel well enough to be tested	0	3 (12.0)	0	2 (10.5)	1 (12.5)	1 (4.6)	0	10 (37.0)	0	1 (5.3)	0	1 (6.7)
I think being tested for HIV is too stressful	0	1 (4.0)	0	0	0	2 (9.1)	0	0	0	1 (5.3)	0	0
Worried about confidentiality	0	0	0	0	0	0	0	1 (3.7)	0	0	0	0.7)
I do not want to know if I have HIV	0	0	0	0	0	0	0	0	0	0	0	0
I plan to be tested in the future	0	2 (8.0)	0	1 (5.3)	0	2 (9.1)	0	5 (18.5)	0	0	0	2 (13.3)
I am scared that I have HIV	0	0	0	0	0	0	0	0	0	0	0	0
Don’t know	0	0	0	0	0	0	0	0	0	0	0	1 (6.7)
Not provided	3 (100.0)	4 (16.0)	6 (75.0)	4 (21.1)	5 (62.5)	0	3 (100)	0	0	0	0	0
REASONS FOR DECLINING HCV TESTING, # (%)	ACCEPTED HIV AND/OR HCV TESTING	DECLINED HIV AND/OR HCV TESTING	ACCEPTED HIV AND/OR HCV TESTING	DECLINED HIV AND/OR HCV TESTING	ACCEPTED HIV AND/OR HCV TESTING	DECLINED HIV AND/OR HCV TESTING	ACCEPTED HIV AND/OR HCV TESTING	DECLINED HIV AND/OR HCV TESTING	ACCEPTED HIV AND/OR HCV TESTING	DECLINED HIV AND/OR HCV TESTING	ACCEPTED HIV AND/OR HCV TESTING	DECLINED HIV AND/OR HCV TESTING
	*n = 3*	*n = 25*	*n = 8*	*n = 19*	*n = 8*	*n = 22*	*n = 3*	*n = 27*	*n = 10*	*n = 19*	*n = 14*	*n = 15*
I do not believe that I am at risk for HCV	0	15 (60.0)	0	12 (63.2)	0	18 (81.8)	0	11 (40.7)	0	17 (89.5)	0	10 (66.7)
I do not feel well enough to be tested	0	3 (12.0)	0	2 (10.5)	0	2 (9.1)	0	10 (37.0)	0	1 (5.3)	0	1 (6.7)
I think being tested for HCV is too stressful	0	1 (4.0)	0	0	0	0	0	0	0	1 (5.3)	0	0
Worried about confidentiality	0	0	0	0	0	0	0	1 (3.7)	0	0	0	0
I do not want to know if I have HCV	0	0	0	0	0	0	0	0	0	0	0	0
I plan to be tested in the future	0	2 (8.0)	0	1 (5.3)	0	2 (9.1)	0	5 (18.5)	0	0	0	3 (20.0)
I am scared that I have HCV	0	0	0	0	0	0	0	0	0	0	0	0
Don’t know	0	0	0	0	0	0	0	0	0	0	0	1 (6.7)
Not provided	3 (100)	4 (16.0)	8 (100.0)	4 (21.1)	8 (100.0)	0	3 (100)	0	10 (100.0)	0	14 (100.0)	0

**Table 4:** Participant-reported HIV/HCV Risk Factors in each Pilot Randomized Controlled Trial.

	*pRCT 1 (n = 55)*				*pRCT 2 (n = 60)*				*pRCT 3 (n = 58)*			
	CDC CONTROL CONDITION VIDEO		PHCI VIDEO		CDC CONTROL CONDITION VIDEO		HIV/HCV COUNSELOR-DELIVERED PHCI		PHCI VIDEO		HIV/HCV COUNSELOR-DELIVERED PHCI	
	ACCEPTED HIV AND/OR HCV TESTING	DECLINED HIV AND/OR HCV TESTING	ACCEPTED HIV AND/OR HCV TESTING	DECLINED HIV AND/OR HCV TESTING	ACCEPTED HIV AND/OR HCV TESTING	DECLINED HIV AND/OR HCV TESTING	ACCEPTED HIV AND/OR HCV TESTING	DECLINED HIV AND/OR HCV TESTING	ACCEPTED HIV AND/OR HCV TESTING	DECLINED HIV AND/OR HCV TESTING	ACCEPTED HIV AND/OR HCV TESTING	DECLINED HIV AND/OR HCV TESTING
	*n = 3*	*n = 25*	*n = 8*	*n = 19*	*n = 8*	*n = 22*	*n = 3*	*n = 27*	*n = 10*	*n = 19*	*n = 14*	*n = 15*
***HIV only risk factors***												
Sexual intercourse with HIV infected partner	0	0	0	0	0	0	0	0	1	0	0	0
Women who have sex with MSM	0	1	0	0	0	0	0	0	0	1	0	0
Sexual intercourse without a condom	3	23	6	13	7	18	3	21	9	16	11	11
History of sexually transmitted infection	0	2	3	1	2	5	1	2	0	1	3	1
***HCV only risk factors***												
Organ transplant before 1992	0	0	0	0	0	1	0	0	0	0	0	0
Receipt of blood transfusion before 1992	0	1	1	1	0	1	0	1	0	0	2	0
Receipt of blood factors before 1987	0	0	0	0	0	0	0	0	0	0	0	0
Sex with an HCV infected person	0	0	0	0	0	0	0	0	0	0	0	0
History of dialysis	0	2	0	0	1	1	0	1	0	0	0	0
Born to an HCV infected mother	0	0	0	0	0	0	0	0	0	1	0	0
Incarceration history	1	5	1	4	1	3	0	0	2	2	3	1
Mother to child HCV transmission	0	0	0	0	0	0	0	0	0	0	0	0
Illicit drug inhalation	0	2	1	2	4	0	2	3	1	3	3	0
Unregulated tattooing	0	0	0	2	0	1	0	1	0	0	2	0
Non-sterile piercing	0	0	0	0	0	0	0	0	0	0	0	0
Sexual contact with HCV infected partner	0	0	0	0	0	0	0	0	0	0	0	0
***HIV & HCV risk factors***												
Injection drug use	0	0	0	1	0	0	0	0	0	0	0	0
MSM	1	0	1	0	0	0	1	1	1	0	0	0

Key: pRCT (pilot randomized, controlled trial), CDC (Centers for Disease Control and Prevention, PHCI (persuasive health communication intervention), MSM (men-who-have-sex-with-men).

**Table 5:** Participant Assessments of the Intervention Received in each Pilot Randomized Controlled Trial.

	*pRCT 1*						*pRCT 2*						*pRCT 3*					
	CDC CONTROL CONDITION VIDEO (n = 28)			PHCI VIDEO (n = 27)			CDC CONTROL CONDITION VIDEO (n = 30)			HIV/HCV COUNSELOR-DELIVERED PHCI (n = 30)			PHCI VIDEO (n = 29)			HIV/HCV COUNSELOR-DELIVERED PHCI (n = 29)		
FEED BACK FACTORS	ACCEPTED HIV AND/OR HCV TESTING	DECLINED HIV AND/OR HCV TESTING	*p-VALUE*	ACCEPTED HIV AND/OR HCV TESTING	DECLINED HIV AND/OR HCV TESTING	*p-VALUE*	ACCEPTED HIV AND/OR HCV TESTING	DECLINED HIV AND/OR HCV TESTING	*p-VALUE*	ACCEPTED HIV AND/OR HCV TESTING	DECLINED HIV AND/OR HCV TESTING	*p-VALUE*	ACCEPTED HIV AND/OR HCV TESTING	DECLINED HIV AND/OR HCV TESTING	*p-VALUE*	ACCEPTED HIV AND/OR HCV TESTING	DECLINED HIV AND/OR HCV TESTING	*p-VALUE*
	*n = 3*	*n = 21*		*n = 8*	*n = 15*		*n = 8*	*n = 22*		*n = 3*	*n = 27*		*n = 10*	*n = 19*		*n = 14*	*n = 15*	
***Convincing***																		
Yes	3 (12.5)	14 (58.3)	0.53	7 (30.4)	9 (39.1)	0.35	8 (26.7)	12 (40.0)	0.03	2 (6.7)	21 (70.0)	0.99	9 (31.0)	10 (34.5)	0.10	14 (48.3)	7 (24.1)	<0.00
No	0	7 (29.2)		1 (4.4)	6 (26.1)		0	10 (33.3)		1 (3.3)	6 (20.0)		1 (3.5)	9 (31.0)		0	8 (27.6)	
***Motivating***																		
Yes	3 (12.5)	14 (58.3)	0.53	7 (30.4)	9 (39.1)	0.35	7 (23.3)	10 (33.3)	0.09	2 (6.7)	23 (76.7)	0.43	8 (27.6)	7 (24.1)	0.05	12 (41.4)	7 (24.1)	0.05
No	0	7 (29.2)		1 (4.4)	6 (26.1)		1 (3.3)	12 (40.0)		1 (3.3)	4 (13.3)		2 (6.9)	12 (41.4)		2 (6.9)	8 (27.6)	
***Respectful***																		
Yes	3 (13.6)	19 (82.6)	0.99	8 (34.8)	13 (56.5)	0.53	6 (20.7)	17 (58.6)	0.99	3 (10.0)	26 (86.7)	0.99	10 (34.5)	16 (55.2)	0.53	14 (48.3)	11 (37.9)	0.10
No	0	1 (4.4)		0	2 (8.7)		2 (6.9)	4 (13.8)		0	1 (3.3)		0	3 (10.3)		0	4 (13.8)	

Key: pRCT (pilot randomized, controlled trial), CDC (Centers for Disease Control and Prevention, PHCI (persuasive health communication intervention).

## References

[1] Merchant RC, Seage GR, Mayer KH, Clark MA, DeGruttola VG, Becker BM. Emergency department patient acceptance of opt-in, universal, rapid HIV screening. Public Health Rep. 2008; 123 Suppl 3: 27–40. DOI: 10.1177/00333549081230S305 PMC2567017 19172704

[2] Merchant RC, Clark MA, Langan TJt, Seage GR 3rd, Mayer KH, DeGruttola VG. Effectiveness of increasing emergency department patients’ self-perceived risk for being human immunodeficiency virus (HIV) infected through audio computer self-interview-based feedback about reported HIV risk behaviors. Acad Emerg Med. 2009; 16(11): 1143–1155. DOI: 10.1111/j.1553-2712.2009.00537.x 20053235 PMC3173950

[3] Merchant RC, Clark MA, Langan TJt, Mayer KH, Seage GR 3rd, DeGruttola VG. Can computer-based feedback improve emergency department patient uptake of rapid HIV screening? Annals of emergency medicine. 2011; 58(1 Suppl 1): S114–119 e111–112. DOI: 10.1016/j.annemergmed.2011.03.035 21684389 PMC3205940

[4] Merchant RC, Baird JR, Liu T, Taylor LE, Montague BT, Nirenberg TD. Brief intervention to increase emergency department uptake of combined rapid human immunodeficiency virus and hepatitis C screening among a drug misusing population. Acad Emerg Med. 2014; 21(7): 752–767. DOI: 10.1111/acem.12419 25125271 PMC4135533

[5] Merchant RC, Clark MA, Santelices CA, Liu T, Cortes DE. Efficacy of an HIV/AIDS and HIV testing video for Spanish-speaking Latinos in healthcare and non-healthcare settings. AIDS and behavior. 2015; 19(3): 523–535. DOI: 10.1007/s10461-014-0889-6 25179540 PMC4346556

[6] Czarnogorski M, Brown J, Lee V, The Prevalence of Undiagnosed HIV Infection in Those Who Decline HIV Screening in an Urban Emergency Department. AIDS Res Treat. 2011; 2011: 879065. DOI: 10.1155/2011/879065 21738860 PMC3124124

[7] Merchant RC, Hernandez D, Estrela D, Fernandez E, Clark MA, Carey MP. Development and refinement of a persuasive health communication intervention to persuade adult emergency department patients to be screened for HIV and hepatitis C. Sage Open. 2021; 11(3). DOI: 10.1177/21582440211047588

[8] Andrews RM, **United States**. Agency for Healthcare Research and Quality. Serving the uninsured: safety-net hospitals, 2003. Rockville, MD: Agency for Healthcare Research and Quality; 2007.

[9] Harrington NG. Persuasive health message design. In: Nussbaum J, ed. Oxford research encyclopedia of communication. New York: Oxford University Press; 2016. DOI: 10.1093/acrefore/9780190228613.013.7

[10] Janz NK, Becker MH. The Health Belief Model: a decade later. Health Educ Q. 1984; 11(1): 1–47. DOI: 10.1177/109019818401100101 6392204

[11] Witte K Putting the fear back into fear appeals: The extended parallel process model. Communication Monographs. 1992; 59(4): 329–349. DOI: 10.1080/03637759209376276

[12] Kahneman D, Tversky A. Prospect theory: an analysis of decision under risk. Econometrica. 1979; 47(2): 263–292. DOI: 10.2307/1914185

[13] Salovey P, Schneider TR, Apanovitch AM. Message framing in the prevention and early detection of illness. In: Dillard JP, Pfau M, eds. The persuasion handbook: Developments in theory and practice. Sage. 2002; 391–406. DOI: 10.4135/9781412976046.n20

[14] Merchant RC, Freelove SM, Langan TJt, The relationship of reported HIV risk and history of HIV testing among emergency department patients. Postgrad Med. 2010; 122(1): 61–74. DOI: 10.3810/pgm.2010.01.2100 PMC3172990 20107290

[15] Pringle K, Merchant RC, Clark MA. Is self-perceived HIV risk congruent with reported HIV risk among traditionally lower HIV risk and prevalence adult emergency department patients? Implications for HIV testing. AIDS patient care and STDs. 2013; 27(10): 573–584. DOI: 10.1089/apc.2013.0013 24093811 PMC3837562

[16] Merchant RC, DeLong AK, Liu T, Baird JR. Factors Influencing Uptake of Rapid HIV and Hepatitis C Screening Among Drug Misusing Adult Emergency Department Patients: Implications for Future HIV/HCV Screening Interventions. AIDS and behavior. 2015; 19(11): 2025–2035. DOI: 10.1007/s10461-015-1103-1 26036465 PMC4600425

[17] Zillmann D, Brosius H-B. Exemplification in communication: the influence of case reports on the perception of issues. Mahwah, NJ: L. Erlbaum Associates; 2000.

[18] Bandura A Social foundations of thought and action: A social cognitive theory. Prentice-Hall; 1986.

[19] National Center for HIV/AIDS, Centers for Disease Control and Prevention. Act Against AIDS Campaign Materials. U.S. Department of Health & Human Services. https://www.cdc.gov/actagainstaids/campaigns/hssc/materials.html. Published 2016. Accessed March 31, 2017.

[20] National Center for HIV/AIDS, Centers for Disease Control and Prevention. Viral Hepatitis - Hepatitis C Information - Patient Education Resources. U.S. Department of Health & Human Services. https://www.cdc.gov/hepatitis/hcv/patienteduhcv.htm. Published 2015. Accessed March 31, 2017.

[21] Ubhayakar ND, Lindsell CJ, Raab DL, Risk, reasons for refusal, and impact of counseling on consent among ED patients declining HIV screening. The American journal of emergency medicine. 2011; 29(4): 367–372. DOI: 10.1016/j.ajem.2009.10.005 20825802 PMC3000887

